# *Mycobacterium interjectum* causing submandibular lymphadenitis in a child

**DOI:** 10.1099/acmi.0.000552.v3

**Published:** 2024-02-08

**Authors:** Sallie Lin, Connie Trieu

**Affiliations:** ^1^​ Department of Pediatrics, University of Florida, Gainesville, Florida, USA; ^2^​ Division of Pediatric Infectious Diseases, University of Florida, Gainesville, Florida, USA

**Keywords:** *Mycobacterium interjectum*, antibiotic, susceptibilities, Nontuberculous mycobacteria

## Abstract

Nontuberculous mycobacteria (NTM) commonly manifest as cervical lymphadenitis in immunocompetent children. Only a few species, such as *Mycobacterium avium complex* (MAC), cause infection in children. With recent advances in gene sequencing, *Mycobacterium interjectum* has been identified as a rare cause of adenitis in children, with at least ten cases reported since 1993. Curative treatment for NTM lymphadenitis, particularly when caused by MAC, usually involves complete surgical excision of the affected lymph nodes. This case report highlights successful treatment of submandibular lymphadenitis caused by *M. interjectum* in a paediatric patient, despite multi-drug resistance *in vitro*.

## Data summary

All data associated with this work is reported within the article.

## Introduction

Nontuberculous mycobacteria (NTM), also known as environmental mycobacteria, are acid-fast bacteria that are widespread in nature and commonly present as cervical lymphadenitis in healthy children. In immunocompromised children, NTM can manifest as disseminated infections. While NTM are found in soil, food, water and animals, tap water is known to be the major reservoir for NTM. Although many are exposed to NTM in the environment, it is unknown why only some individuals become infected. Infections are thought to be introduced via skin abrasions, where wounds become contaminated with soil or water. For cervical lymphadenitis, oropharyngeal mucosa is the presumed entry site for NTM [1].

Of the approximately 200 NTM species identified, there are only a few species of NTM known to infect humans [1] *Mycobacterium avium complex*, *M. abscessus* and *M. fortuitum* are among some of the NTM species commonly known to cause cervical lymphadenitis in children [1]. *Mycobacterium interjectum* is a recently identified species of mycobacteria, and limited data is available with regards to its susceptibility profile and treatment. As reported in the literature, *M. interjectum* infections have generally presented as lung manifestations in adults while causing cervical adenitis in the paediatric population [2]. The first case of paediatric cervical lymphadenitis caused by *M. interjectum* was reported in 1993 [3, 4]. Since then, at least ten cases of lymphadenitis in immunocompetent children caused by *M. interjectum* have been reported in the literature [2], with isolates expressing multi-drug resistance *in vitro* [3–6]. This case report highlights successful treatment of NTM lymphadenitis caused by *M. interjectum* in a paediatric patient, adding to our knowledge surrounding treatment for this rare mycobacterium species.

## Case

A 22-month-old female with no significant past medical history was admitted to the hospital for persistent right submandibular swelling for 3 weeks. Patient had been afebrile without systemic symptoms. She attended daycare and recently had a right ear infection. She had exposure to a cat 1 week prior to the onset of facial swelling. Prior laboratory work by her paediatrician showed mildly elevated erythrocyte sedimentation rate (ESR) 30 mm/h and C-reactive protein (CRP) 17.18 mg l^−1^. Complete blood count (CBC) was without leukocytosis. Complete metabolic panel (CMP) was unremarkable. Epstein–Barr virus (EBV) DNA quantitative PCR was equivocal. *Bartonella henselae* serologic tests were negative. During the first week of swelling, a soft tissue ultrasound showed a 3.1×1.6 cm group of lymph nodes in the right mandibular region, as well as bilateral enlarged cervical lymph nodes. The right submandibular swelling was stable over the initial 2 weeks; however, swelling and tenderness worsened over the third week. A second mass developed, and the overlying skin became erythematous and purplish in discolouration. Failure of amoxicillin-clavulanate and trimethoprim-sulfamethoxazole courses prompted presentation to the emergency room.

In the emergency room, laboratory work was obtained, with results as follows: CBC unremarkable, repeat *Bartonella henselae* serologic tests negative, EBV viral capsid antigen (VCA) IgM negative, and EBV VCA IgG positive at 339 U ml^−1^. CRP was 7.16 mg l^−1^, and ESR was 31 mm/h. Ultrasound of her right mandible visualized a hypoechoic area and an enlarged 1.6×1 cm lymph node with increased vascularity. No discrete drainable fluid was seen. Due to concern for suppurative lymphadenitis, the patient was started on intravenous antibiotics with azithromycin and clindamycin. During her hospitalization, the right submandibular masses progressed and became fluctuant and mobile. Incision and drainage (*I* and *D*) was performed, and findings were significant for necrotic right submandibular lymph nodes. Purulent drainage and necrotic material were sent for cultures, including mycobacteria growth indicator tube (mgit) and Lowenstein–Jensen medium. The patient was discharged home to complete 5 days of azithromycin and 7 days of clindamycin.

The patient initially did well with resolution of right submandibular swelling. However, 1–2 weeks after *I* and *D*, her swelling and erythema returned in the same locations. Central necrotic scabs also developed. An additional 7 days of clindamycin was prescribed by her primary care provider, and the patient was referred to Paediatric Surgery and Paediatric Infectious Diseases for further evaluation and management. Acid-fast bacilli culture from the *I* and *D* had growth after 8 days. Via HAIN line-probe assay for further identification, the organism was identified as *Mycobacterium interjectum*. As listed in Table 1, the organism was resistant to ciprofloxacin (MIC 8 ug ml^−1^), doxycycline (MIC >8 ug ml^−1^), and rifampin (MIC 4 ug ml^−1^) but susceptible to amikacin (MIC 8 ug ml^−1^), clarithromycin (MIC 1 ug ml^−1^), linezolid (MIC 8 ug ml^−1^), moxifloxacin (MIC 0.5 ug ml^−1^), rifabutin (MIC 1 ug ml^−1^) and trimethoprim/sulfamethoxazole (MIC 1/19 ug ml^−1^). The patient subsequently underwent complete surgical excision of her lymph nodes for curative treatment with no further signs of recurrence.

**Table 1.** Comparison of *Mycobacterium interjectum* susceptibilities

Article	Age	Susceptible (MIC)	Resistant (MIC)
This case	22-month-old	Amikacin (8 ug ml^−1^) Clarithromycin (1 ug ml^−1^) Linezolid (8 ug ml^−1^) Moxifloxacin (0.5 ug ml^−1^) Rifabutin (1 ug ml^−1^) Trimethoprim/sulfamethoxazole (1/19 ug ml^−1^)	Ciprofloxacin (8 ug ml^−1^) Doxycycline (>8 ug ml^−1^) Rifampin (4 ug ml^−1^)
De Baere *et al*. [5]	3-year-old	Clarithromycin (1 ug ml^−1^*) Rifabutin (40 ug ml^−1^*) Cycloserine (60 ug ml^−1^†) Ethionamide (10 ug ml^−1^†) Ofloxacin (4 ug ml^−1^†)	Rifampin (40 ug ml^−1^*) Isoniazid (0.2 ug ml^−1^*) Ethambutol (2 ug ml^−1^*) *para*-aminosalicylic acid (0.5 ug ml^−1^*) Streptomycin (4 ug ml^−1^*) Kanamycin (6 ug ml^−1^†) Capreomycin (10 ug ml^−1^†)
Springer *et al*. [3]	18-month-old	Rifampin	Isoniazid Streptomycin Ethambutol

*Lowenstein-Jensen.

†Middlebrook 7H11 agar.

## Discussion

In immunocompetent children, lymphadenitis secondary to *M. interjectum* has been reported only in a handful of cases since 1993 [2], occurring in Germany, Belgium, Sweden, Australia and Italy [3–6]. Cervical lymphadenitis is thought to be caused by entry of NTM from the environment via route of oropharyngeal mucosa [1, 2]. In most reports, infection manifested as unilateral cervical lymphadenitis, with ages ranging from approximately 18 months to 3 years old [4]. After excision, cultures from surgical material grew in approximately 2–4 weeks [4], as expected from this slow-growing mycobacteria [2]. While ‘rapidly growing’ mycobacteria can be grown and identified in 3–7 days, ‘slow-growing’ mycobacteria requires weeks [1].

As a newer species of mycobacteria, the prevalence of *M. interjectum* is likely underreported due to limitations in identification through phenotypic and biochemical characteristics, as *M. interjectum* shares similarities to other NTM organisms [2, 4]. Advancements in sequencing, particularly 16S rRNA, have made accurate identification of *M. interjectum* possible [2, 4, 5]. In this case, HAIN line-probe assay, which is based on the PCR method, was used to identify the organism.

Complete surgical excision is considered the optimal treatment for NTM lymphadenitis [1], as the natural history of NTM lymphadenitis without surgical intervention is typically slow resolution with a high risk for spontaneous drainage and fistula formation [3, 4]. Consistent with previous case reports, complete surgical excision was curative for our patient with lymphadenitis caused by *M. interjectum*.

In cases where complete surgical excision is not an option or is incomplete, antibiotics may be considered [1]. Empiric therapy with clarithromycin or azithromycin in addition to rifampin or rifabutin and/or ethambutol [1] may not be reasonable treatment for *M. interjectum*. Clinical isolates of *M. interjectum*, for which limited susceptibility data are available, have been reported to express multi-drug resistance [5, 6]. A two- to three-drug treatment regimen of NTM should be guided by species identification and drug susceptibility testing [1]. Few studies report MIC susceptibilities for *M. interjectum,* as seen in Table 1.

## Conclusion

In conclusion, NTM infection should be considered as a possible infectious aetiology for paediatric cases of cervical/submandibular lymphadenitis unresponsive to routine antibiotics. Current recommendation for treatment involves complete surgical resection. In cases where antimicrobial treatment is elected, with or without surgical intervention, drug-susceptibility testing is needed to help guide antibiotic choices. Only a few cases report the susceptibilities of *M. interjectum*, and this case report aims to provide additional susceptibility data to help guide other providers in choosing empiric therapy for *M. interjectum* infection while awaiting sensitivities.

## References

[1] Kimberlin DW, Barnett ED, Lynfield R, Sawyer MH, American Academy of Pediatrics (2021). Red Book: 2021–2024 Report of the Committee on Infectious Diseases, Committee on Infectious Diseases, American Academy of Pediatrics.

[2] Sotello D, Hata DJ, Reza M, Satyanarayana R, Arunthari V (2017). Disseminated *Mycobacterium interjectum* infection with bacteremia, hepatic and pulmonary involvement associated with a long-term catheter infection. Case Rep Infect Dis.

[3] Springer B, Kirschner P, Rost-Meyer G, Schröder KH, Kroppenstedt RM (1993). *Mycobacterium interjectum*, a new species isolated from a patient with chronic lymphadenitis. J Clin Microbiol.

[4] Tuerlinckx D, Fauville-Dufaux M, Bodart E, Bogaerts P, Dupont B (2010). Submandibular lymphadenitis caused by *Mycobacterium interjectum*: contribution of new diagnostic tools. Eur J Pediatr.

[5] De Baere T, Moerman M, Rigouts L, Dhooge C, Vermeersch H (2001). *Mycobacterium interjectum* as causative agent of cervical lymphadenitis. J Clin Microbiol.

[6] Rose M, Kitz R, Mischke A, Enzensberger R, Schneider V (2004). Lymphadenitis cervicalis due to *Mycobacterium interjectum* in immunocompetent children. Acta Paediatr.

